# Treatment of Alzheimer’s Disease: Beyond Symptomatic Therapies

**DOI:** 10.3390/ijms241813900

**Published:** 2023-09-09

**Authors:** Francesca R. Buccellato, Marianna D’Anca, Gianluca Martino Tartaglia, Massimo Del Fabbro, Elio Scarpini, Daniela Galimberti

**Affiliations:** 1 Department of Biomedical, Surgical and Dental Sciences, University of Milan, 20122 Milan, Italy; 2 Fondazione IRCCS Ca’ Granda, Ospedale Maggiore Policlinico, 20122 Milan, Italy

**Keywords:** Alzheimer’s disease (AD), therapy, drug, disease-modifying therapy (DMT), monoclonal antibody (mAb), small molecules

## Abstract

In an ever-increasing aged world, Alzheimer’s disease (AD) represents the first cause of dementia and one of the first chronic diseases in elderly people. With 55 million people affected, the WHO considers AD to be a disease with public priority. Unfortunately, there are no final cures for this pathology. Treatment strategies are aimed to mitigate symptoms, i.e., acetylcholinesterase inhibitors (AChEI) and the N-Methyl-D-aspartate (NMDA) antagonist Memantine. At present, the best approaches for managing the disease seem to combine pharmacological and non-pharmacological therapies to stimulate cognitive reserve. Over the last twenty years, a number of drugs have been discovered acting on the well-established biological hallmarks of AD, deposition of β-amyloid aggregates and accumulation of hyperphosphorylated tau protein in cells. Although previous efforts disappointed expectations, a new era in treating AD has been working its way recently. The Food and Drug Administration (FDA) gave conditional approval of the first disease-modifying therapy (DMT) for the treatment of AD, aducanumab, a monoclonal antibody (mAb) designed against Aβ plaques and oligomers in 2021, and in January 2023, the FDA granted accelerated approval for a second monoclonal antibody, Lecanemab. This review describes ongoing clinical trials with DMTs and non-pharmacological therapies. We will also present a future scenario based on new biomarkers that can detect AD in preclinical or prodromal stages, identify people at risk of developing AD, and allow an early and curative treatment.

## 1. Introduction

The estimated number of patients with Alzheimer’s disease (AD) dementia is about 50 million worldwide. This number will increase to 150 million by 2050 [1,2]. As amyloid beta (Aβ) deposition can precede cognitive decline, a preclinical stage with neuropathologic changes, positive biomarkers of AD pathology, including Aβ and tau, can last many years or decades without any symptoms. Thus, it can be estimated that there is an even more significant number of persons with an increased risk of progression to cognitive impairment [2,3]. This global scenario urges the need to design new treatment strategies to prevent the onset and progression of AD. AD neuropathology is characterized macroscopically by cerebral cortical thinning and atrophy, where synapsis and neuronal loss contribute to the atrophy [4]. From a microscopic point of view, the hallmarks of AD pathology are neuritic Aβ plaques and neurofibrillary tangles [4]. Misfolded Aβ and tau proteins accumulate in the AD brain in a neurodegenerative cascade leading to cognitive decline and dementia [4,5,6,7,8,9]. Aβ plaques are derived from the β-amyloid precursor protein (APP), which undergoes an alternative proteolytic pathway, in which Aβ peptides are generated by endoproteolytic cleavage of APP by β- and γ-secretase [4,5,6,7,8,9]. The Aβ protein exists in various conformational states, including soluble monomers, soluble aggregates (oligomers, protofibrils), and insoluble fibrils and plaque [4,5,6,7,8,9]. These different forms may mediate different pathogenic events in AD [10]. Among these various forms, soluble Aβ aggregates are more toxic than monomers or insoluble fibrils; thus, reducing these soluble Aβ aggregates could represent a practical treatment approach in the early stages of AD [10,11,12].

AD pathology is a proteinopathy caused by protein misfolding and aggregation [13]. This process is common to other neurodegenerative diseases, such as Parkinson’s disease, Huntington’s disease, light chain amyloidosis, and spongiform encephalopathies [14,15]. A common characteristic of these diseases is the conversion of normally soluble proteins into insoluble fibrillar aggregates [13]. Each proteinopathy is characterized by different fibrillar aggregates, such as amyloid-β (Aβ) plaques and neurofibrillary tangles of tau in AD, Lewy bodies of α-α-synuclein (αSyn) in PD, or amyloid deposits of an immunoglobulin light chain in AL [16]. The protein homeostasis system typically prevents amyloid accumulation, which declines in ageing [17]. The Aβ oligomer hypothesis, supported by in vitro, in vivo, and ex vivo models, points to the toxic Aβ oligomers rather than amyloid plaques as key players in AD pathogenesis [4,5,6,7]. Elevated levels of oligomers have been associated with AD, as they are found in AD brains and are considered the most toxic and pathogenic form of Aβ [4,5,6,7,18]. In this regard, experimental data supports a pathogenic mechanism in which AD neuropathology and cognitive loss are the consequences of the Aβ oligomers toxicity on neurons. Low molecular weight Aβ oligomers, which are the most aqueously diffusible, may mediate disruption of neurite integrity and synaptic plasticity [19]. Recently, the physicochemical origin of oligomer toxicity has been deeply studied [16]. Pairs of toxic and non-toxic oligomers have been discovered for Aβ42, alpha-synuclein and other proteinopathies. Common physicochemical properties in the different pairs have been found, including a higher fraction of solvent-exposed hydrophobic residues, which demonstrate a high affinity for biological membranes, resulting in an ability to disrupt them and the capacity to induce cellular dysfunction [16]. In conclusion, solvent-exposed hydrophobic residues appear to be a key shared property of toxic oligomers [16]. Oligomers can also contribute to AD pathogenesis via neuron-to-neuron spreading or by generating new fibrils following neuronal uptake. Aβ oligomers exert their toxicity, promoting a rapid decrease in membrane expression of memory-related receptors, reducing spine density, and promoting synaptic deterioration in cultures of hippocampal neurons [19]. The biological mechanisms that ultimately lead to neuronal cell death and neurodegeneration include membrane perturbation, intracellular Ca2+ influx mediated by N-Methyl-D-aspartate (NMDA) and Adenosine Monophospate (AMP) receptors, mitochondrial dysfunction, reactive oxygen species (ROS) production, lipid peroxidation, an increased caspase-3 response, and aberrant protein−protein interactions [20]. It is worth noting that in the AD drug development pipeline, mAbs are designed to target Aβ oligomers, as demonstrated by Lecanemab. In addition, a promising class of small molecules is aminosterols, which prevent the binding of toxic oligomers Aβ40, Aβ42, and αSyn to cells, thereby eliminating their toxicity [21].

Tau, a microtubule-associated protein, is highly expressed in neurons and has been implicated in the pathogenesis of AD and other neurodegenerative diseases called “tauopathies” [19,20,22,23,24,25]. Tauopathies are neurodegenerative diseases characterized by a common pathological hallmark: aggregated tau deposition in the brain [26,27]. The current hypothesis in AD pathogenesis takes into consideration the early accumulation of Aβ species and plaques, which precedes the spreading of tau, neuronal loss, and clinical manifestations over two decades [22,26,27,28]. The collective body of knowledge suggest that Aβ accumulation represents an upstream pathophysiological event and may function as a trigger/facilitator of downstream molecular pathways, including tau misfolding, tau-mediated toxicity, accumulation in tangles, and tau spreading that leads to cortical neurodegeneration [24,26,27,29]. Indeed, evidence shows that tau is significantly elevated in the cerebrospinal fluid (CSF) of AD patients, and its increase is an early event before the onset of the clinical signs [22]. Furthermore, both total and phosphorylated tau (p-tau) are increased in the CSF of AD patients and can predict the progression of the disease [22,30,31]. In this scenario, the accumulation of Aβ has been considered the primary injury, and therapeutic approaches have been targeted towards Aβ removal. However, the subsequent tau pathology and tau-mediated neurodegeneration recently suggested that tau pathology can progress independently of Aβ accumulation [32]. Tau-targeted therapies as alternatives to Aβ-targeted treatments have recently emerged as potential strategies for treating AD patients [3].

AD disease-modifying strategies are designed to target abnormal Aβ aggregates, clear, and delay or prevent the formation of aggregates [33]. Historically, AD disease-modifying strategies beyond symptomatic medications have relied on (a) anti-Aβ production and aggregation agents, decreasing the synthesis of Aβ, such as β- and γ-secretase; (b) neutralizing or removing the toxic aggregate or misfolded forms of Aβ by immunotherapy; (c) anti-tau protein drugs [33,34].

The present review will resume the history of AD-modifying treatment, which started in 1999 with the first preclinical evidence of the effectiveness of vaccination against Aβ in mice until the development of many disease-modifying drugs in recent years [35]. Furthermore, we will also analyze the recent literature regarding mAbs and other biologic DMTs, which are designed against AD and are currently being tested in clinical trials. Details of such clinical trials were obtained from publications or listings on https://clinicaltrails.gov. We will also discuss some challenges and open questions arising from current clinical trials [36]. From a future perspective, cell therapies for AD are foreseen, and now, eight stem cell trials for AD are ongoing [3]. We will also discuss the need for an ideal peripheral biomarker for the early diagnosis of AD that permits the treatment of patients in the very first stages of the disease.

## 2. Immunotherapy Overview

Since the beginning, in the history of AD treatment approaches, active immunotherapy or passive immunotherapy against Aβ have shown the potential of altering Aβ deposition and has been extensively studied in animal models of AD [37]. The first preclinical evidence of the effectiveness of vaccination against Aβ in mice came from the study of Schenk et al., where immunization of PDAPP transgenic mice that overexpress mutant human APP essentially prevented the development of β-amyloid-plaque formation, neuritic dystrophy, and astrogliosis [35]. The work of Schenk et al. provided evidence that the reduction in β-peptide deposition observed in PDAPP mice after the vaccination was likely caused by Fc-mediated phagocytosis [35]. In addition, microglia were colocalized with β-peptide within plaques as demonstrated by confocal microscopy [35]. Importantly, microglia/monocytes were found almost entirely near the few remaining plaques. Furthermore, the levels of APP were not altered by the immunization treatment, nor was there any evidence of toxic effects in the treated animals [35].

From this first study, the possibility that immunization with Aβ may be effective in preventing and treating AD led to a trial of an Aβ vaccination in AD patients. AN-1792, a vaccine with fibrillar human Aβ42 in conjunction with a novel adjuvant QS-21 [38]. Clinical studies of AN-1792 reached phase 2a, in which 372 patients with probable AD (mild to moderate) were enrolled [38]. The study was discontinued due to aseptic meningoencephalitis symptoms in 6% of the patients [38]. However, in a subset of immunized patients, slower rates of cognitive decline were observed [38]. The long-term effects of active immunization with the same Aβ42 synthetic peptide were studied, and the results after 6 years showed the clearance of amyloid plaques but no evidence of increased survival rate or improvement of severe dementia [39]. These facts have accelerated the development of passive immunization approaches and safer vaccines. Various antibodies against different parts of the Aβ peptide were designed, Bapineuzumab, Solanezumab, and Ponezumab, and tested in human clinical trials in patients with overt symptoms with no success [40]. Other approaches included secretase enzyme modulation by alpha secretase activators and beta and gamma secretase inhibitors (Semagacestat, Avagacestat, and Verubecestat) [41,42,43]. Bristol-Myers Squibb halted development of γ-secretase inhibitor, Avagacestat, in 2012 after the analysis of two phase 2 trial results that demonstrated the worsening in cognition and a greater incidence of adverse effects in treated people compared to those on a placebo. These trial ends followed the termination of the Semagacestat phase 3 trial by Eli-Lilly (2010) and the complete abandonment of the γ-secretase inhibitors in the AD drug development pipeline. Verubecestat, developed to lower the brain levels of Aβ, is a small molecule taken orally that targets beta-site amyloid precursor protein cleaving enzyme 1 (BACE-1) and inhibits BACE1 ability to cleavage APP and form Aβ [44]. However, this small molecule was dismissed after the failure of the phase 3 clinical trial without a previous real phase 2 trial to establish the long-term safety of verubecestat [43,44]. Verubecestat, administered in patients with mild-to-moderate AD, was not beneficial and increased adverse effects [45]. It is worth mentioning that the period of 2017–2018 marks the termination of various trials and the abandonment of the investment in drugs against AD by Pfizer and other companies [46].

## 3. Failure of Many Clinical Trials: Why?

Phase III trials should be initiated with a robust foundation, usually coming from the results of phase II trials (safety, tolerability, dose-finding), considering a wide number (often thousands) of patients and a treatment duration ≥ 1.5 years [46]. Sometimes potential issues arise from too early initiated phase III trials, such as drug safety, incorrect choice of drug dose, inappropriate evaluation of biomarkers data, or post hoc subgroup analysis [46].

Data on phase III clinical trials showed that failure of clinical trials is mainly due to the following limitations: (1) dementia was too severe, i.e., neuron damage too extensive to see any effect, thus patients with very mild dementia should be treated rather than moderately–severely impaired; (2) no biomarker was required as eligibility criteria for inclusion; (3) lack of informative outcome measures or surrogate biomarkers of clinical response [46,47,48,49]. To overcome the above difficulties, European Medicine Agency (EMA) and Food and Drug Administration (FDA) have issued guidelines focus on the usefulness of biomarkers in AD clinical trials in the context of drug development: (1) At the diagnosis, to determine the AD stage; (2) To select the patients eligible for treatment; (3) At prognosis, to estimate the progression of the disease; (4) To predict the clinical response; (5) To identify the intended and non-intended effects of therapy [50]. Furthermore, FDA and EMA guidelines have pointed to the standardization of the use of biomarkers in clinical trials to ensure consistency and replicability [51]. The preclinical stage of AD, with neuropathologic changes and positive biomarkers of AD pathology, including Aβ and tau, can last many years or decades without any symptoms. Identifying these patients is paramount, as biomarkers may also demonstrate therapy effects and serve as the basis for accelerated approval of new drugs. In later-stage patients, biomarkers may provide evidence for a drug with clinical benefits. From this, however, the question arises of which biomarkers are most suitable.

According to the NIA-AA research framework, the biomarkers for AD are Aβ deposition (A), pathologic tau (T), and neurodegeneration [AT(N)] [52]. Recently, it has been established that Aβ pathology (A) can be determined using either Aβ PET or CSF Aβ interchangeably [52]. Instead, the use of tau PET and CSF p-tau interchangeably for tau pathology (T) seems to be more complex [52]. Finally, according to the NIA-AA research framework, markers of neurodegeneration (N), such as hippocampal volume and/or cortical thickness of temporoparietal regions, can be determined using structural magnetic resonance imaging (MRI) [52]. In this regard, it is worth noting that emerging fluid biomarkers of neurodegeneration have recently been studied [53]. Indeed, blood-based biomarkers for AD could pave the way in supporting the diagnosis of AD in clinical practice and in improving the development of clinical trials for the earliest stages of AD [53,54,55].

## 4. State of the Art of AD Disease-Modifying Treatments (DMT)s

### 4.1. mAbs Anti-Aβ and Anti-Tau

**Figure 1:**
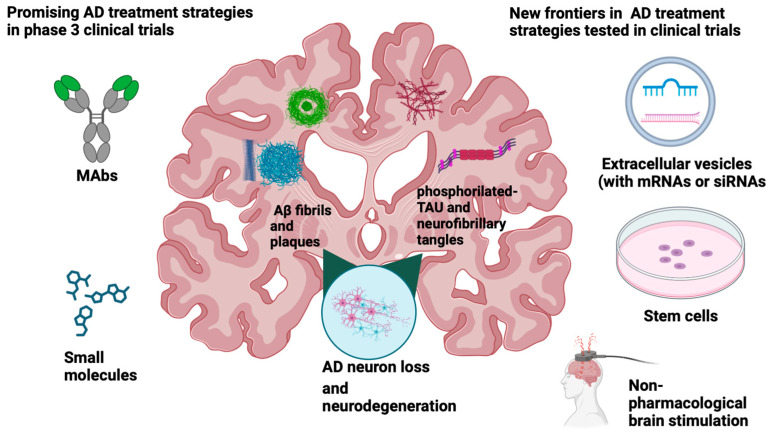
Summary of Alzheimer’s disease (AD) treatment strategies in clinical trials. Created with Biorender.com.

As previously anticipated, passive immunotherapy is considered the most effective approach to preventing and treating AD (see Figure 1) [36,37]. Currently, the AD drug development pipeline includes 36 agents in phase III, 25% are DMT-biologics, and 42% are DMT small molecules. Seven (19% of the total DMT in the pipeline) of these agents are mAbs designed to bind to different parts of the aminoacidic sequence of Aβ (see Table 1) [3]. There is also an anti-tau mAb and a receptor agonist as biologic agents included in the review of Cummings et al. [3]. Potential immunotherapy against AD and tested in phase III clinical trials also include tertomotide, a 16-amino-acid peptide comprising a sequence from the human enzyme telomerase reverse transcriptase (TERT).

#### 4.1.1. Aducanumab

Aducanumab has been tested in a phase II study, demonstrating its biological effect in terms of Aβ removal via PET with amyloid tracer [56]. In light of this evidence, two large phase III studies were started, with three arms (high dose, low dose, placebo) [57,58,59,60]. However, in March 2019, Eisai and Biogen announced the termination of all ongoing aducanumab trials due to the failure of interim analysis [61,62]. On 22 October 2019, Biogen declared that the interim futility analysis was wrong and that subsequent analysis of a more extensive data set showed that EMERGE had met its primary endpoint regarding the high, but not the low dose [62,63]. This led to the accelerated approval of Aducanumab by the FDA for the treatment of mild cognitive impairment due to AD and mild AD dementia on 7 June 2021. The approval of the first mAb against AD was given without a definitively proven clinical benefit based on its effects on AD biomarkers [47,62,64,65].

Based on the results of phase 3 clinical trials, aducanumab can effectively lower amyloid PET ligand signals to the levels of controls in many individuals with early AD but controversial data on possible cognitive and functional improvement in AD patients treated with Aducanumab have arisen [40]. In addition, Aducanumab, as with other mAbs, can induce radiographic features known as AD-related imaging abnormalities with edema (ARIA-E) or hemorrhage (ARIA-H) [66]. Consequently, the updated use recommendations of Aducanumab emphasize the importance of detecting past medical conditions that may predispose the patient to ARIA or may increase the likelihood of ARIA complications [47]. Given the controversial results of clinical trials and the data available on Aducanumab, post-approval studies are ongoing. Aducanumab is now tested in a phase 3b Open-Label, Multicenter extension study (NCT04241068) called EMBARK [3]. The purpose of the study was to evaluate the long-term safety and tolerability of Aducanumab in participants with AD who had previously participated in Aducanumab studies (PRIME, ENGAGE, EMERGE, EVOLVE) and received Aducanumab or placebo at the time of the announcement of early termination. The study will be completed in October 2023. ENVISION (NCT 05310071) is a phase 3b/4 Multicenter, Randomized, Double-Blind, Placebo-Controlled, Parallel-Group Study ending in 2025. The ENVISION trial will help to shed light on the challenging questions on the effectiveness of Aducanumab and will potentially strengthen the link between amyloid clearance and cognitive improvement.

#### 4.1.2. Donanemab

Donanemab (N3pG) is a humanized mAb developed from mouse mE8-IgG2a [67]. This mAb is specific for the N-terminally, pyroglutamate-modified Aβ proteins found in amyloid plaques and aids plaque removal through microglial-mediated phagocytosis. Donanemab is supposed to target deposited plaque and a clear amyloid burden from the brain rather than prevent the deposition of new plaques or the growth of existing plaques. The mE8 antibody has been reported to reduce deposited amyloid plaques in mice without inducing microhemorrhages [67]. Three different trials testing Donanemab (NCT04437511, NCT05026866, NCT05508789) are ongoing [3]. TRAILBLAZER-ALZ2 (NCT04437511) is a phase 3, double-blind, placebo-controlled study to evaluate the safety and efficacy of donanemab in participants aged 60–85 years with early symptomatic AD (MCI or mild dementia due to AD) with the presence of confirmed amyloid and tau deposition with biomarkers. The trial enrolled 1736 participants across eight countries, selected based on cognitive assessments in conjunction with amyloid plaque imaging and tau staging by PET imaging, and ended in April 2023. On 4 May 2023, Lilly announced positive results for this phase 3 study, as treatment significantly slowed cognitive decline on the primary outcome of the integrated AD Rating Scale (iADRS) by 40% and improved all secondary clinical endpoints [68,69]. Donanemab significantly slowed the clinical progression in individuals with early symptomatic AD and amyloid and tau pathology. The results of TRAILBLAZER-ALZ 2 show that diagnosing and treating people earlier in the course of AD may lead to greater clinical benefit [68,69]. The company will apply for FDA approval in the next few months. The other two trials with Donanemab (NCT05026866, NCT05508789) will be completed by 2027.

#### 4.1.3. Gantenerumab

Gantenerumab is a fully human antibody that has a high affinity for Aβ fibrils [70]. It was selected because it can bind both the N-terminal and central amino acids of Aβ [70]. This antibody can disassemble and degrade amyloid plaques by recruiting microglia and activating phagocytosis [71,72]. Gantenerumab preferentially interacts with the aggregated form of Aβ and was developed for subcutaneous administration [71,72,73]. On 8 October 2021, the Breakthrough Therapy Designation was granted by the FDA for the treatment of AD. However, GRADUATE 1 and 2 trials failed to meet the primary endpoints, and Roche discontinued all trials with Gantenerumab in January 2023 [74].

#### 4.1.4. Lecanemab

Lecanemab (BAN2401, NCT03887455) is a humanized mAb that preferentially targets soluble aggregated Aβ, with activity across oligomers, protofibrils, and insoluble fibrils being tested in persons with early AD. Eisai/Biogen has applied to the FDA for a marketing license and received approval on January 2023. Unlike with aducanumab, the companies filed directly under the accelerated-approval pathway using phase 2b data, which showed drastic plaque reduction [75]. The BAN2401-G000-201 trial did not meet the 12-month primary endpoint. However, prespecified 18-month Bayesian and frequentist analyses demonstrated a reduction in brain amyloid and a consistent reduction in clinical decline across several clinical and biomarker endpoints [75]. A phase 3 study (Clarity AD) in early AD has recently ended. This 18-month, multicenter, double-blind, phase 3 trial involving persons 50 to 90 years of age with early AD (mild cognitive impairment or mild dementia due to AD disease) demonstrated that Lecanemab reduced markers of amyloid in early AD. Further, it resulted in moderately less decline in measures of cognition and function than the placebo at 18 months but was associated with adverse events [76]. The study, in conclusion, showed that longer trials are still needed to determine the efficacy and safety of Lecanemab in early AD [77].

#### 4.1.5. Remternetug

Remternetug (N3pG-Aβ mAb, previously LY3372993) is an investigational mAb. It recognizes a pyroglutamated form of Aβ aggregated in amyloid plaques, similar to Donanemab. In August 2022, Lilly started phase 3, called TRAILRUNNER-ALZ1 (NCT05463731), a randomized, double-blind, placebo-controlled study to evaluate the safety and efficacy of remternetug in participants with early symptomatic AD [78]. Initially, 600 participants will be enrolled in the double-blind treatment period. Participants will receive remternetug or a placebo administered via subcutaneous injection or intravenous infusion. An extended study period will permit all participants to receive the drug after the study, as remternetug recipients will cross over to placebo, and placebo recipients to the drug. The study plans to end in October 2026.

#### 4.1.6. Solanezumab

Solanezumab is an mAb targeting soluble Aβ. In three completed phase 3 clinical trials (EXPEDITION, EXPEDITION2, EXPEDITION3), solanezumab did not significantly reduce the decline in cognition or function (e.g., activities of daily living and community involvement) in patients who had received a clinical diagnosis of mild-to-moderate AD [79]. In a more recent phase 3 trial (NCT02008357), solanezumab failed to meet primary and secondary outcomes, as the drug did not slow cognitive decline in preclinical AD or reduce the risk of progression to symptomatic AD [80]. The same drug is being tested in a phase II/III Multicenter Randomized, Double-Blind, Placebo-Controlled Platform Trial of Potential Disease Modifying Therapies Utilizing Biomarker, Cognitive, and Clinical Endpoints in Dominantly Inherited AD (NCT01760005) [3].

#### 4.1.7. E2814

E2814 is a humanized mAb that recognizes an HVPGG peptide epitope in the microtubule-binding domain near the mid-domain of tau [76]. The latter is essential in AD, as it is involved in the seeding and spread of pathological tau tangles. The antibody binds extracellular tau, prevents cell-to-cell propagation of pathogenic species, and is involved in the clearance of tau aggregates by microglia. As N-terminally targeted anti-tau antibodies have shown no efficacy in clinical trials, more potent mid-region antibodies have been designed to interfere with the propagation of pathogenic aggregates of tau [81]. The drug is currently being tested in a phase II/III Multicenter Randomized, Double-Blind, Placebo-Controlled Platform Trial of Potential Disease Modifying Therapies Utilizing Biomarker, Cognitive, and Clinical Endpoints in Dominantly Inherited AD (NCT05269394). This study will be completed in 2027.

#### 4.1.8. Semaglutide

Semaglutide is a glucagon-like peptide-1 (GLP-1) receptor agonist (RA) used in the treatment of type 2 diabetes and obesity, where it effectively lowers glucose levels, body weight, and risk of cardiovascular disease. Glucagon-like peptide-1 (GLP-1) has been recognized as a potent stimulator of insulin secretion and a key regulator of energy homeostasis. Diabetes is associated with an increased risk of dementia. This association may in part be due to systemic mitochondrial dysfunction that is common to these pathologies [82]. Mitochondrial dysfunction is a significant feature of AD and may play a fundamental role in its pathogenesis [82]. Therefore, targeting glucose metabolism and insulin resistance could be an important step in over-coming mitochondrial dysfunction and cholesterol metabolism failures in ageing or AD [83]. In this view, an appropriate treatment approach for AD may include an early target, such as Aβ and tau, and a later and continuous restoration of brain metabolic function to prevent cognitive function [84]. GLP1-RAs is known for its neuroprotective action, and more recent results have emerged, showing a potential benefit in people with AD [85,86,87]. Novo Nordisk evaluated GLP-1 data from preclinical models, real-world studies involving patient registry and insurance claims databases, and post hoc analysis of data from three large cardiovascular outcome trials of semaglutide and liraglutide and reported a 53% reduction in the risk of developing dementia in people with Type 2 diabetes who took a GLP-1 agonist compared to placebo. Based on this analysis, Novo Nordisk announced the development of semaglutide, which is currently being investigated in two trials (EVOKE, NCT04777396, and EVOKE Plus NCT04777409) to assess effects in early AD. These studies will be completed in October 2026.

#### 4.1.9. Tertomotide

Tertomotide or GV1001, originally designed as a cancer vaccine, is a 16 amino acid peptide comprising a sequence from the human enzyme of the human telomerase reverse transcriptase catalytic subunit (hTERT) [88]. Telomerase is a ribonucleotide enzyme that maintains telomeres and confers cancer cell immortality. It is mostly expressed in cancer but not in normal cells so that TERT peptides can serve as effective cancer-targeting antigens. Experimental data also suggest that GV1001 exerts unexpected biological activities. These independent-telomeric functions of hTERT are associated with cellular proliferation, stem cell mobilization, anti-apoptotic, and antioxidant effects through mitochondrial stabilization, transcriptional regulation, and effects on the Wnt signaling pathway [89,90]. In rat neuronal stem cells, GV1001 blocked Aβ oligomer-induced toxicity and increased the survival of neuronal cells exposed to oxidative stress [91,92]. In a completed phase 2 study conducted in Korea, GV1001 showed significant improvement from the baseline of Severe Impairment Battery score at Week 24 and demonstrated a clinically acceptable safety profile in patients with moderate to severe AD [93]. NCT05303701, a phase 3 study, was registered to start in January 2023 with the aim of enrolling Korean patients with moderate to severe AD and will be completed in April 2026.

### 4.2. Small Molecules

The complexity in the pathogenesis and in the interactions with related genes and proteins makes the research of drugs against AD that can have a long-term effect or even provide a cure challenging [94]. Indeed, the best knowledge of the pathogenic mechanisms underpinning the etiology of AD lays the foundation for the research and development of new potential treatment strategies. Thus, alongside the historical pathological hallmarks of AD, Aβ aggregates, hyperphosphorylated Tau protein, and neural loss, other kinds of pathogeneses and mechanisms have been proposed, and new possible druggable targets are emerging in the landscape of pharmacological agents. Although the genetic susceptibility, numerous findings support the involvement in AD pathogenesis of other mechanisms such as inflammation [95], imbalance among production and removal of ROS [96], mitochondrial dysfunction [82], in addition to the β-amyloid cascade [97] and the deficiency of central cholinergic neurotransmitters [98] on which are based most of anti-AD drugs so far approved. Currently, the small molecules marketed for AD mitigate symptoms and attenuate disease progression, acting as cognitive enhancers. In particular, out of only five drugs approved, four of them are AchEI to stimulate cholinergic neurotransmission (Tacrine, retired due to hepatoxicity, Donepezil, Galantamine, Rivastigmine), while Memantine acts as the antagonist of NMDA receptors in order to activate them [99,100]. Since their counterbalancing action on the cholinergic imbalance of the disease, these classes of drugs attenuate symptoms, providing temporary relief from mild to moderate to severe AD [101]. Even if they can delay cognitive decline in AD, they do not establish a cure for the disease.

Looking at phase III clinical trials, there is a progressive interest in DMT small molecules [3]. Fifteen agents of the 36 in phase III are small molecules and represent 42% of the total DMT agents [3] (See Figure 1 and Table 2).

#### 4.2.1. Hydralazine

The efficacy of hydralazine (NCT 04842552) against AD is being assessed in a randomized clinical trial in early stage AD patients who take one of the AChEIs, donepezil, rivastigmine, or galantamine.

Hydralazine, an FDA-approved antihypertensive, has been chosen for its recently discovered anti-neurodegenerative efficacy based on his action on antioxidative stress mechanisms, such as activation of the Nrf2 signaling pathway, increase of mitochondria respiration capacity and adenosine triphosphate production, activation of autophagy, which facilitates intracellular aggregate clearance [102].

#### 4.2.2. Omega 3

Recent findings showed significant correlations between long-chain omega-3 levels and blood–brain barrier (BBB) integrity and cognition, providing evidence of a possible mechanism by which omega-3 may contribute to brain health [103]. Furthermore, lower omega-3 fatty acid levels are correlated to reduced brain blood flow to regions important for learning, memory, depression, and dementia [104]. The goal of the study (NCT 02719327) is to evaluate the efficacy of a purified form of the omega-3 fatty acid, eicosapentaenoic acid EPA on MRI, CSF, and cognitive biomarkers for AD in 150 cognitively healthy veterans aged 50–75 years with increased risk of AD. The study will end in September 2023.

#### 4.2.3. Metformin

Regarding the role of glucose metabolism in AD and the association of Type 2 Diabetes with AD, medicament metformin (NTC04098666) is in phase III [3]. Metformin is an insulin sensitizer medicine, a first-line treatment, and a widely prescribed oral treatment for type 2 diabetes. Glucose metabolism amelioration in the Central Nervous System (CNS) is supposed to improve the cognitive decline of early and late amnestic Mild Cognitive Impairment patients or prevent the cognitive decline in obese people [3,105,106]. The trial will end in March 2026.

#### 4.2.4. AGB101

Since 2003, FDA has approved no new small molecules, despite many long and expensive trials [107]. Therefore, in the current landscape of treatment research, drugs that are usually known for the treatment of other diseases appear. This is the case of the anti-seizure AGB101 (low-dose extended-release Levetiracetam) preferentially prescribed for epilepsy in older people due to its lack of drug–drug interactions [108]. Furthermore, seizures are more frequent in aged people with AD than in those without dementia [109]. This is probably linked to general neuronal loss in the brain of AD patients. AGB101, assessed in a phase III clinical trial (NCT03486938) in MCI patients, acts by modulating the synaptic protein SV2A. In the early stages of AD, the drug is supposed to reduce neuronal hyperactivity Aβ-induced fighting the memory impairment and slowing the disease progression [110]. The study, HOPE4MCI, lead sponsor AgeneBio, involved 164 participants between MCI and prodromal AD and ended on November 2022. The FDA has not approved AGB101 since its safety and efficacy have not been established.

#### 4.2.5. Anavex2-73

The drug Blarcamisine (Anavex 2-73) has the same intent of neuroprotection but with a different mechanism of action. It is a mixed ligand of the sigma-1 receptor (σ1R) and the M2 muscarinic receptor. The σ1R, primarily present in many tissues, is broadly expressed in CNS cells [111]. Interestingly, the levels of this receptor are maintained or increased in the normal-aged brain of animal models [112,113], and σ1R increases with healthy ageing have also been reported in human studies [114]. Instead, in the very early phases of AD, a significant loss of σ1R occurs in the brain [115,116]. In addition, σ1R is involved in controlling protein tau phosphorylation [117]. In preclinical studies, ANAVEX 2-73 showed significant results reverting cognitive impairment in AD mice models, suggesting anti-amnesic and neuroprotective action [118]. This amino tetrahydrofuran derivative is undergoing a further phase IIb/III clinical trial (NCT04314934) to evaluate the effects on the safety and efficacy of daily treatment of this drug. The trial, titled Open Label Extension Study for Patients with Early AD enrolled in Study ANAVEX2-73-AD-004, is an interventional study still in the recruiting phase sponsored by Anavex Life Sciences Corporation (New York, NY, USA).

#### 4.2.6. Fosfogonimenton

The lack of approved therapies and the complicated pathological processes involved have led to the research of different targets and approaches to treat AD. Therefore, neurotrophic factors and their receptors are novel therapeutic strategies being followed. In this context, hepatocyte growth factor (HGF), highly active in most CNS cell types, is a critical neurotrophic factor that, upon binding the mesenchymal epithelial transition factor (MET) receptor tyrosine kinase, elicits mitogenic, mitogenic, and morphogenic functions [119]. The HGF/MET system promotes neural survival and regeneration, inducing several proneuronal and precognitive processes. Existing literature supports the neurotrophic and neuroprotective role of HGF/MET signaling in several mouse models of different neurological disorders, including Aβ-induced cognitive impairment mice, where the HGF/MET implementation reduced disease progression and restore function [120,121]. These findings are in keeping with clinical literature showing low levels of MET protein in the brains of AD patients and the MET gene as the most downregulated by transcriptome analysis [122,123]. It follows that a compound, such as the Fosfogonimenton (ATH-1017, NDX-1017), by positively modulating the HGF/MET system, could aid neurodegeneration and promote neuronal health and function. Indeed, the Fosgo-AM, the active metabolite of Fosfogonimenton, in dementia animal models has already demonstrated the capability to stimulate the HGF/MET system activating synaptic plasticity and restoring cognitive function. Furthermore, the prodrug and clinical candidate developed later, Fosfogonimenton, confirmed in vivo its positive effects, reverting cognitive deficits [124]. The safety and efficacy of ATH-1017, renamed NDX-1017, is being evaluated in a phase III clinical trial (NCT04488419) sponsored by Athira Pharma. A randomized, double-blind, placebo-controlled study in patients with mild to moderate AD is still ongoing.

#### 4.2.7. Masitinib

The effort to find an innovative non-amyloid-based compound acting on neuroinflammation could be another treatment option since the growing body of evidence supports the involvement of the neuroimmune system, mast cells (MCs), and microglia in AD onset and progression [125,126]. Indeed, if the CNS homeostasis balance is perturbed, microglial cells try to redress the equilibrium being activated. If the imbalance persists, microglia are pushed to a stronger activation state termed priming. Primed microglia can stimulate the release of Aβ and tau protein with the following formation of neuritic plaques and neurofibrillary tangles, as well as reducing the production of neurotrophic factors [125]. Masitinib is a tyrosine kinase inhibitor that interferes with the survival, migration, and activity of MCs and microglia, showing a neuroprotective effect in neurodegenerative diseases and concentrating on the CNS at a therapeutically relevant amount [127,128]. In preclinical studies, Masitinib exerted a synaptoprotective effect by inhibiting MCs and restoring normal spatial learning performance [129]. The positive results of a previous phase IIb/III study (AB09004; NCT01872598) revealed the positive effects of Masitinib, which slowed down cognitive impairment in mild to moderate AD patients [130]. A confirmatory pivotal study in phase III (NCT05564169) with a randomized, double-blind, parallel group is to date ongoing to assess the treatment effect of Masitinib as an add-on therapy with cholinesterase inhibitor and/or memantine in patients with mild to moderate AD sponsored by AB Science.

#### 4.2.8. NE3107

As mentioned earlier, primed microglia represent the main player in AD neuroinflammation [131]. Multiple factors and pathways are indeed engaged in the pathophysiological activation of microglia. Among them, MAPK signaling is activated in the early stages of immune activation [132]. Extracellular signal-regulated kinase (ERK) proteins 1 and 2 are part of the MAPK family. In preclinical AD mouse models, ERK1 and ERK2 represent the stronger upregulated phosphoproteins in the MAPK family and are increased in post-mortem human AD brains [133]. This means that changes in ERK protein expression may regulate pro-inflammatory immune activation in AD. Furthermore, Aβ induces the ERK-dependent serine phosphorylation of the insulin receptor substrate (IRS) in astrocytes, inhibiting its ability to transduce insulin signals to downstream targets [134]. Consequently, the failure of glucose use leads to neuronal death and insulin resistance (IR) in the brain. This probably explains why up to 81% of AD patients also suffer from impaired glucose tolerance (IGT) and Type 2 diabetes (T2D) [135]. Therefore, ERK inhibition would attenuate both the inflammation and the insulin response. The compound NE3107 (formerly HE3286) is a derivative of β-androstenetriol, an adrenal sterol derived from the human metabolome, is blood–brain permeable, binds ERK1/2, and acts as an anti-inflammatory insulin sensitizer [136]. In neurodegeneration murine models, NE3107 has been shown to reduce inflammation-driven ERK, the primed microglia, and amyloid precursor protein, and it selectively inhibits NF-kB [137,138]. Positive results of the phase II trial (NCT05227820) were presented in December 2022 at the Clinical Trial in Alzheimer’s Disease (CTAD) annual conference. NE3107-treated patients showed a cognition improvement and a reduction of CSF p-TAU levels, with no adverse events observed [139]. To date, NE3107 has been assessed in a phase III trial in mild and moderate AD (NCT04669028), whose details have been published in 2021 funded by BioVie Inc. (Carson City, NV, USA) [140]. This multicenter, randomized, placebo-controlled trial, whose baseline data were presented in June as a poster at the 83rd American Diabetes Association Annual Meeting, was designed to confirm the efficacy and safety of the agent in patients with probable AD [141]. The trial is now enrolling patients and expanding to 45 sites.

#### 4.2.9. Nilotinib

The Aβ treatment failures have prompted the investigation of new pathways to target and other brain regions beyond the temporal lobe, including the hippocampal region. Autophagy undoubtedly represents a crucial process in neural development and homeostasis through which harmful and/or redundant macromolecules are cleared thanks to lysosomal pathways [142,143]. Indeed, the clearance of Aβ deposits exploits mainly the autophagic pathways, as well as an autophagy dysfunction, which is associated with the increased release of β-amyloid [144,145]. It follows that impairments in autophagy processes are indeed involved in AD pathogenesis, especially since pathological aβ species interfere with their own clearance, triggering a vicious circle between impaired autophagy and protein aggregation in AD. Therefore, enhancing autophagy could be an alternative strategy in AD treatment. Nilotinib, a c-Abl tyrosine kinase inhibitor already approved for the treatment of chronic myeloid leukemia, has been shown to prevent degeneration in murine models of AD [146,147]. In particular, Nilotinib was able to reduce Aβ and phospho-c-Abl levels, restoring autophagic flow in Tg2576 dopaminergic neurons of several brain regions [148]. Another work investigated the role of Nilotinib in brain mitochondrial dysfunction, highlighting how this drug could ameliorate mitochondrial function in the brain astroglia of an AD mouse model [149]. In a previous phase II study, Nilotinib was safe and well tolerated in mild to moderate AD, penetrating the BBB and achieving pharmacologically relevant levels in the CSF [150]. These data supported the design of a phase 3 study, named NILEAD, sponsored by KeifeRx, LLC, (Mclean, VA, USA) randomized, double-blind, placebo-controlled to evaluate the efficacy and safety of Nilotinib BE (bioequivalent) in early AD (NCT05143528). This study is not yet in the recruitment phase and will involve around 1275 subjects.

#### 4.2.10. Melatonin

Melatonin, a hormone mainly synthesized and secreted in the pineal gland, decreases during ageing and even more in AD patients, likely leading to cognitive impairments [151,152]. In addition, melatonin inhibits the β-fibrillogenesis with neuroprotective and antioxidant activities in vitro and in vivo AD models [153,154]. On this basis, the Piromelatine (or Neu-P11) agent acting as an agonist of melatonin and serotonin receptors has been demonstrated to improve memory and neuronal and cognitive impairments in preclinical studies [155]. This multimodal drug was developed for treating insomnia that AD patients exhibit severely, and it is associated with worsened cognitive and memory capacities [156]. In the ReCOGNITION trial, agomelatine was evaluated for its efficacy in treating AD itself rather than for treating sleeplessness in mild AD patients. Indeed, the primary endpoint used the cNTB, a computerized version of the neuropsychological test battery. The secondary endpoint evaluated AD with functional, clinical, global, and neuropsychiatric scales, such as the ADAS-cog14, in addition to the safety and sleep quality index, such as the PSQI. The results showed no significant differences among mild AD patients versus the placebo. Later, a GWAS analysis performed on 107 patients enrolled in the same trial identified six single nucleotide polymorphisms (SNPs) on the 2q12 chromosome linked to agomelatine responsiveness. The 27% of GWAS samples analyzed carried the SNPs, and the relative patients improved at cNTB but worsened at ADAS-cog14 and PSQI under piromelatine administration. On the other hand, the non-carriers improved significantly with piromelatine compared to the placebo on the ADAS-Cog14 and PSQI with piromelatine [157]. This means that the efficacy of Neu-P11 in mild AD patients could be predicted by profiling their polymorphisms. That is why the subsequent clinical trial registered from Neurim Pharmaceuticals Ltd. (NCT05267535) includes only mild AD subjects, “non-carrier” of those SNPs. This multicenter study, randomized, delayed start, double-blind, parallel-group, placebo-controlled, is enrolling patients with mild dementia due to AD to assess the efficacy and safety of agomelatine 20 mg.

#### 4.2.11. Simufilam

The major knowledge and research on the molecular mechanisms of AD pathogenesis led to the study with interest of the α7 nicotinic acetylcholine receptor (α7nAChR) that binds with high-affinity the soluble amyloid β1-42(Aβ42) triggering the protein tau hyperphosphorylation [158,159]. Investigating this binding, Wang et al. discovered that the scaffold protein Filamin A (FLNA) interacted with the α7nAChR when it bound Aβ42 in mouse models of AD [160]. In particular, the resulting complex, activating ERK and JNK1 kinases, which hyperphosphorylated tau protein, prevented the tau microtubule stabilization and contributed to neuronal dysfunction and degeneration [160,161]. The relevance of FLNA in the β-amyloid pathogenic cascade is even more evident when ex-vivo post-mortem brains were incubated, or mice were treated with its inhibitor compounds, such as Simufilam [160,161]. Indeed, in the AD brains, Simufilam binds with high-affinity FLNA stronger than native FLNA, reducing the affinity of Aβ42 with its receptor to the point where Aβ42 is displaced from its binding site. The tau hyperphosphorylation is prevented, allowing microtubule stabilization [162]. This proposed mechanism in preclinical models encouraged us to also pursue Simufilam treatment in AD patients. Cassava Sciences, Inc., in November 2021, began two phase III clinical trials with Simufilam (PTI-125), both still ongoing. The first one randomizes 750 participants with mild to moderate AD (NCT04994483) to evaluate the safety and efficacy of PTI-125 100 mg tablets. The second one (NCT05026177) will recruit 1083 subjects with mild to moderate AD to evaluate the safety and efficacy of two doses of Simufilam, 50mg or 100mg, versus placebo. Although the judicial troubles have involved the company regarding alleged research results manipulated and then denied, Cassava Sciences, Inc. announced an open-label long-term extension for phase III trials, optional (NCT05575076) to evaluate the safety and tolerability of Simufilam 100 mg tablets in participants with mild to moderate AD. Enrollment by invitation will include 1600 participants.

#### 4.2.12. ALZ-801

Not only are monoclonal antibodies developed against β-amyloid, but there is also a small molecule agent, named ALZ-801, that prevents neurotoxic Aβ formation with no plaque interaction [163]. ALZ-801 is a prodrug of tramiprosate with the same major metabolite, 3-sulfopropanoic acid (3-SPA), physiologically present in the human adult brain and in the CSF of subjects with cognitive deficits due to AD and other neurodegenerative disorders [164].

Furthermore, the 3-SPA byproduct showed a marked anti-Aβ oligomer activity inhibiting Aβ42 aggregation and consequently toxic Aβ oligomers formation, a key step in AD pathogenesis and progression [164]. Interestingly, tramiprosate showed the highest efficacy linked to the presence of the ε4 allele of the apolipoprotein E gene (APOE4), a critical risk factor for AD onset [165]. In mild AD patients, the clinical benefits of tramiprosate treatment were more pronounced in homozygosis (APOE4/4) carriers rather than in heterozygosis (APOE4/3) or non-carriers (APOE3/3), in whom there was no benefit [166]. The clinical trial of this compound failed, while Alzheon Inc. licensed ALZ-801, actually in phase III clinical trial (NCT04770220). The study multicenter, randomized, double-blind, placebo-controlled, assesses the efficacy, safety, and biomarker effects of ALZ-801 in 300 APOE4/4 subjects with early AD. The study has not yet begun recruitment.

#### 4.2.13. Hydromethylthionine Mesylate

Tau aggregation is a promising target for AD DMT, as it is a hallmark pathology of AD that correlates with younger AD symptom onset and can progress independently from amyloid pathology [32,167]. Hydromethylthionine mesylate (HMTM) is a tau aggregation inhibitor designed to reduce tau aggregation pathology in AD [168]. Lucidity (NCT03446001) is a phase 3, randomized, double blind, placebo-controlled, outpatient trial to evaluate the safety, efficacy, and tolerability of HMTM monotherapy in participants with mild cognitive impairment (MCI) to moderate AD. The phase 3 clinical trial included an initial 12-month period in which participants received orally administered HMTM 16 mg/day, HMTM 8 mg/day, or methylthioninium chloride (MTC) given twice weekly as a control, followed by an additional 12-month blinded, open-label extension during which time all participants received HMTM 16 mg/day. Results from a prespecified analysis of the phase 3 LUCIDITY trial show a statistically significant reduction in blood neurofilaments (NfL) for people receiving 16 mg/day of HMTM compared to controls. Neurofilaments and tau proteins are essential for neuronal structure and function in the brain. In this regard, NfL concentration reduction in blood demonstrated that the drug targets tau pathology and reduces neurodegeneration without causing ARIAs. These recent results were presented at the 2023 meeting of the Alzheimer’s Association International Conference (AAIC) in Amsterdam, The Netherlands.

## 5. Other Therapeutic Approaches: Stem Cells

The lack of effective therapies urges the research of novel approaches for diagnostic tools and therapeutic agents against AD. In this context, stem-cell therapies based on human mesenchymal stem cells (hMSCs) are emerging as a new frontier in the AD treatment horizon (see Figure 1). Indeed, MSCs show peculiar characteristics that make them extremely promising for cellular therapy. First, they can also differentiate into neurons upon specific growth factor exposure [169]. Second, they are less prone to transformation into neoplastic cells [169]. Third, as they do not expose on their surface the MHC-2, they are less immunogenic and challenging to reject from the immune system, especially since they can suppress T cell activation, modulating the immune function with an immunosuppressive effect [169,170]. In AD treatment, the MSCs bone marrow-derived (BM-MSCs) are particularly interesting since they can accelerate microglia activation, reduce amyloid-β deposition and ameliorate cognitive abilities in AD mouse models [171]. Moreover, they can substitute damaged neurons, recovering several impaired functions and differentiating in Schwann cells regenerating neurons [172,173]. Another source of MSCs is represented from adipose tissue, where MSCs adipose tissue-derived (AT-MSCs) can differentiate in cholinergic neurons under specific morphogenic treatments in vitro models [174]. When treated with melatonin, these MSCs attenuate symptoms of AD associated with naïve AT-MSCs [175]. Finally, MSC transplantation improved neurogenesis and synaptic activity, suppressing neuroinflammation in AD murine models [176]. The secretome from MSCs as a potential therapy for neurodegenerative diseases is also worth mentioning. Indeed, the MSC secretome contains numerous bioactive molecules able to attenuate AD symptoms and indirectly contribute to Aβ-amyloid plaque degradation [177]. Extracellular vesicles released by MSCs are part of the secretome, and their usage as therapeutic tools has already been reported in many neurological disease models [178,179]. Their BBB permeability and low immunogenicity certainly make them appealing regarding drug delivery [180,181]. Indeed, MSCs can be engineered to release EVs containing siRNA or miRNA against β-amyloid plaques directly (see Figure 1) [182]. Nowadays, no clinical trials with MSCs have still achieved phase III, but some of them are assessed in phase II studies (NCT02833792, NCT04482413, NCT05233774).

## 6. Non-Pharmacological Interventions

Since we do not have a decisive therapy for the treatment of AD, the best approach seems to be associating pharmacological with non-pharmacological interventions usually tailored to a single patient. These approaches are designed to reduce cognitive decline and improve psychosocial behaviors to slow down neurodegeneration. They can be distinguished into cognitive-oriented interventions, physical exercise interventions, brain stimulation, nutrition supplementations and/or diet restrictions, as well as music therapy and phototherapy [183]. Cognitive-oriented interventions that include stimulation, training, and individualized cognitive rehabilitation, albeit with different specificities, involve the subject’s cognitive abilities through paper and pencil or computer tests and/or ecological activities for compensatory and/or rehabilitation purposes [184]. They aim to stimulate the cognitive reserve as the brain’s ability to actively solve a pathological process through the remodeling of brain networks, allowing the offset of the impairment due to the disease [185]. In cognitive-oriented interventions, the most popular is the Cognitive Stimulation Therapy (CST). This includes multifaceted approaches that do not act specifically on cognitive mechanisms, but using reminiscence therapy and Reality Orientation Therapy (ROT), they aim to enhance general cognitive and social functioning with comparable effectiveness to the AChE inhibitors to slow down cognitive decline [186]. The CST also includes the Validation Therapy based on empathy and the Occupational Therapy (OT) to help the patient regain independence. Furthermore, reminiscence therapy takes advantage of different sensorial stimuli to evoke and retrieve past and recent memories, such as musical and visual stimulation [187]. Actually, the cognitive training approach has achieved phase III in a randomized clinical trial enrolling 7600 adults 65 years of age and older with a primary prevention purpose (NCT03848312). The study is intended to ascertain whether a specific cognitive intervention, speed of processing training (SPT), significantly delays the onset of clinically defined MCI or dementia across three years, based on the results of a previous study, ACTIVE, where reasoning and speed, but not memory, training resulted in improved targeted cognitive abilities for 10 years [188].

Music therapy, phototherapy, and aromatherapy, alone or combined, have resulted in positive outcomes in cognition, emotion, and behavior [189]. Physical exercises are reported to improve cognition and quality of life, and home-based exercises seemed to improve balance and fear of falling and executive functions [190,191,192]. Due to the apparent complexity of performing physical exercises, especially in patients with severe AD, a good strategy could be to work on personal habits to deliver physical interventions, such as regular walking [193]. Another alternative treatment strategy is to target neural circuit dysfunctions, known as parts of AD pathological processes that contribute to the brain network disruption that could cause cognitive impairment [194]. The brain stimulation approaches that include, for example, deep brain stimulation (DBS) or transcranial direct current stimulation (tDCS), use electricity in order to “turn on” or to “turn off specific brain activities acting with invasive tools, as the DBS, or not, as the tDCS [195]. Previously used to reduce tremors, stiffness, and uncontrollable movements in Parkinson’s patients, DBS requires neurosurgery intervention to implant a couple of electrodes into a specific brain area with an internal pulse generator modulating neuronal activity [195,196]. Even if in several murine models, DBS has been shown to improve visual-spatial memory, in a phase II study with 2 years of follow-up on mild AD patients, DBS targeting the fornix was safe and well tolerated but with no clinical benefits [197].

Among the non-invasive brain stimulation techniques, tDCS is the most safe, tolerable and low-cost to administer for a long time at home [198]. This technique induces robust excitability changes using a low direct current within the cortical cortex. Although the number of clinical trials involving tDCS is increasing, none of them has raised phase III. Instead, another non-invasive brain stimulation technique, Transcranial Pulse Stimulation (TPS), is now being used in a phase III study (NCT05983575). This novel ultrasound-based stimulation allows targeting precise regions of interest, and it is reported to improve depression in AD patients [199]. The study is a multicenter, randomized, double-blinded, placebo-controlled phase 3 study comparing a LIPUS-Brain transcranial low-intensity pulsed-wave ultrasound device to a placebo in patients with early AD, and now it is in the enrollment phase.

Finally, dietary approaches in terms of nutrition supplementation, such as combined formulation of specific active ingredients or probiotics, or nutritional guidance, such as fasting intermitting or Mediterranean diet, ameliorate both cognition and function of prodromal and mild AD patients [200,201,202]. Regarding this multimodal approach, there is a very recent phase III study named EVANTHEA, sponsored by Alzheimer’s Prevention and Reversal Project, Inc., to evaluate the effectiveness of a precision medicine treatment approach for early dementia and MCI (NCT05894954). This study is intended to compare a precision medicine approach that involves a combination of medicines, dietary supplements, lifestyle changes, and diagnostics with the standard of care for people with early AD and MCI. A previous pilot study revealed improvement in all outcome cognitive measures and in MRI brain volumetrics [203], supporting the performance of a large randomized clinical trial started in June and not still in the enrollment phase.

Since there is a lack of effective therapy, non-pharmacological interventions represent low-cost and with no side effects tools to ameliorate the quality of life of people with dementia and their caregivers. Indeed, for people with dementia, the caregiver plays a fundamental role in their existence. Therefore, another element that should be considered is caregiver burden, which affects physical and psychological health more than non-caregivers [204]. This means that caregivers should be supported in their complicated care roles as much as AD patients should. In conclusion, the difficult management of AD patients should provide pharmacological and non-pharmacological therapies perfectly tailored and combined with the caregiver’s well-being, mainly to prevent the burden phenomena.

## 7. Future of AD Diagnosis and Treatment

In the new era of AD treatment marked by the availability of mAbs that can modulate disease progression and cognitive decline, the need for the future is the possibility of detecting AD in preclinical or prodromal stages [205]. The next generation of clinical care for patients with AD will need different biomarkers (BMs), such as screening BMs (in elderly non-demented patients) for early referral to memory clinics and proximity BMs to predict symptom development. These ideal BMs should be peripheral and non-invasive (blood/saliva) [206,207]. Screening BMs will be part of a process that aids primary care practitioners in deciding which patients should be referred to a memory clinic. This will also help to reduce the overall clinic and medical system burden and decrease the number of unnecessary referrals and diagnostic procedures [205]. A primary care-based blood screening tool would not be intended as diagnostic but as a gatekeeper to confirm further diagnostic procedures. In this context of use (screening), the negative predicting value (NPV) will be much more important than the positive predictive value (PPV) (i.e., NPV must be ≥90%, PPV should be >50%) to avoid false negatives. This will allow us to refer to memory clinic positive subjects for conventional CSF/PET analysis. The early diagnosis will permit the identification of people at risk instead of patients, and the treatment should be a secondary prevention attempt in subjects at high risk rather than a cure for symptomatic patients.

## Figures and Tables

**Table 1:** Summary of biologic Alzheimer’s disease (AD) Disease Modifying Therapies (DMTs) (mAbs, other biologic agents) in phase 3 clinical trials.

Medication	Mechanism of Action	Clinical Trial Number	Estimated Completion Date/Current Status
Aducanumab	Anti-amyloid mAb directed at plaques and oligomers	NCT04241068 NCT05310071	October 2023 December 2025
Donanemab	Anti-amyloid mAb specific for pyroglutamate modified Aβ plaque proteins	NCT04437511 NCT05026866 NCT05508789	Completed October 2027 April 2027
Gantenerumab	Anti-amyloid mAb directed at plaques and oligomers	NCT04339413 NCT04374253 NCT05256134 NCT05552157	Terminated Terminated Terminated Suspended
Lecanemab	Anti-amyloid mAb directed at protofibrils and plaques	NCT03887455 NCT04468659 NCT05269394 NCT01760005	September 2027 October 2027 October 2027 October 2027
Remternetug	Anti-amyloid mAb recognizing pyroglutamated form of Aβ plaques	NCT05463731	October 2026
Solanezumab	Anti-amyloid mAb targeting soluble Aβ	NCT02008357 NCT01760005	Terminated October 2027
E2814	Anti-tau mAb	NCT05269394	October 2027
**Other biologic agents**			
Semaglutide	glucagon-like peptide-1 receptor agonist	NCT04777396 NCT04777409	
Tertomotide	Vaccine, 16aa peptide from human telomerase reverse transcriptase	NCT05303701	April 2026

**Table 2:** Summary of small molecules in phase 3 clinical trials.

Small Molecules			
Hydralazine hydrocloride	Free radical scavenger	NCT04842552	December 2023
Icosapent ethyl	Purified form of the omega-3 fatty acid eicosapentaenoic acid	NCT02719327	September 2023
Metformin	Insulin sensitizer	NTC04098666	April 2026
AGB101	Anti-seizure medicament	NCT03486938	November 2022, Ended
Blarcamesine (ANAVEX 2-73)	Mixed ligand of the sigma-1 receptor (σ1R) and the M2 muscarinic receptor	NCT04314934	July 2024
Fosfogonimenton (ATH-1017, NDX-1017)	HGF/MET system	NCT04488419	February 2024
Masitinib	Tyrosine kinase inhibitor	NCT05564169	December 2025
NE3107	Derivative of β-androstenetriol	NCT04669028	October 2023
Nilotinib	c-Abl tyrosine kinase inhibitor	NCT05143528	June 2026
Piromelatine	Agonist of melatonin and serotonin receptors	NCT05267535	June 2025
Simufilam	Inhibitor of scaffold protein Filamin A	NCT05575076	July 2027
Valiltramiprosate (ALZ-801)	Prevents neurotoxic Aβ formation with no plaque interaction	NCT04770220	June 2024
Hydromethylthionine mesylate	Tau aggregation inhibitor	NCT03446001	Completed

## Data Availability

Not applicable.
